# Salivary Gland Ultrasonography in Sjögren's Syndrome: A European Multicenter Reliability Exercise for the HarmonicSS Project

**DOI:** 10.3389/fmed.2020.581248

**Published:** 2020-11-23

**Authors:** Alen Zabotti, Sara Zandonella Callegher, Annarita Tullio, Arso Vukicevic, Alojzija Hocevar, Vera Milic, Giacomo Cafaro, Marina Carotti, Konstantina Delli, Orazio De Lucia, Diana Ernst, Francesco Ferro, Angelica Gattamelata, Giuseppe Germanò, Ivan Giovannini, Daniel Hammenfors, Malin V. Jonsson, Sandrine Jousse-Joulin, Pierluigi Macchioni, Simone Parisi, Carlo Perricone, Martin Helmut Stradner, Nenad Filipovic, Athanasios G. Tzioufas, Francesca Valent, Salvatore De Vita

**Affiliations:** ^1^Rheumatology Clinic, Department of Medical Area, Academic Hospital S. Maria della Misericordia, University of Udine, Udine, Italy; ^2^Institute of Epidemiology, Academic Hospital 'Santa Maria della Misericordia', Udine, Italy; ^3^Faculty of Engineering Science, University of Kragujevac, Kragujevac, Serbia; ^4^Bioengineering Research and Development Center (BioIRC), Kragujevac, Serbia; ^5^Department of Rheumatology, University Medical Centre Ljubljana, Ljubljana, Slovenia; ^6^Institute of Rheumatology, Belgrade, Serbia; ^7^Rheumatology Unit, Department of Medicine, University of Perugia, Perugia, Italy; ^8^Department of Radiology, Ospedali Riuniti, Università Politecnica delle Marche, Ancona, Italy; ^9^Department of Oral and Maxillofacial Surgery, University of Groningen, University Medical Center Groningen, Groningen, Netherlands; ^10^Unit of Clinical Rheumatology, Department of Rheumatology and Medical Sciences, Aziende Socio Sanitarie Territoriali (ASST) Centro Traumatologico Ortopedico G. Pini-Centri Traumatologici Ortopedici (CTO), Milan, Italy; ^11^Clinic for Immunology and Rheumatology, Hannover Medical School, Hannover, Germany; ^12^Rheumatology Unit, Department of Clinical and Experimental Medicine, University of Pisa, Pisa, Italy; ^13^Department of Internal Medicine and Medical Specialties, Rheumatology, Sapienza University of Rome, Rome, Italy; ^14^Rheumatology Unit, Department of Internal Medicine, Azienda Ospedaliera Arcispedale Santa Maria Nuova (ASMN), Istituto di Ricovero e Cura a Carattere Scientifico, Reggio Emilia, Italy; ^15^Broegelmann Research Laboratory, Department of Clinical Science, University of Bergen, Bergen, Norway; ^16^Department of Rheumatology, Haukeland University Hospital, Bergen, Norway; ^17^Section for Oral and Maxillofacial Radiology, Department of Clinical Dentistry, University of Bergen, Bergen, Norway; ^18^Rheumatology Department, Centre Hospitalier Universitaire (CHU) de Brest, Univ Brest, Brest, France; ^19^UMR 1227, Lymphocytes B et Autoimmunité (LBAI), Inserm, Univ Brest, Brest, France; ^20^Unit of Rheumatology, Department of General and Specialty Medicine, Città della Salute e della Scienza, Turin, Italy; ^21^Department of Rheumatology and Immunology, Medical University of Graz, Graz, Austria; ^22^Department of Pathophysiology, School of Medicine, National and Kapodistrian University of Athens, Athens, Greece

**Keywords:** Sjögren's syndrome, scoring system, classification criteria, reliability exercise, salivary gland ultrasonography

## Abstract

**Objectives:** Salivary gland ultrasonography (SGUS) is increasingly applied for the management of primary Sjögren's syndrome (pSS). This study aims to: (i) compare the reliability between two SGUS scores; (ii) test the reliability among sonographers with different levels of experience.

**Methods:** In the reliability exercise, two four-grade semi-quantitative SGUS scoring systems, namely De Vita et al. and OMERACT, were tested. The sonographers involved in work-package 7 of the HarmonicSS project from nine countries in Europe were invited to participate. Different levels of sonographers were identified on the basis of their SGUS experience and of the knowledge of the tested scores. A dedicated atlas was used as support for SGUS scoring.

**Results:** Twenty sonographers participated in the two rounds of the reliability exercise. The intra-rater reliability for both scores was almost perfect, with a Light's kappa of 0.86 for the De Vita et al. score and 0.87 for the OMERACT score. The inter-rater reliability for the De Vita et al. and the OMERACT score was substantial with Light's Kappa of 0.75 and 0.77, respectively. Furthermore, no significant difference was noticed among sonographers with different levels of experience.

**Conclusion:** The two tested SGUS scores are reliable for the evaluation of major salivary glands in pSS, and even less-expert sonographers could be reliable if adequately instructed.

## Introduction

Primary Sjögren's syndrome (pSS) is a systemic autoimmune and lymphoproliferative disease, mainly involving the salivary glands (SGs) (1). In pSS, the SGs inflammatory process ultimately results in glandular structural damage (2, 3). Active glandular lesions are characterized by inflammation and lymphoproliferation, with varying degrees of glandular damage by fibrosis, fatty accumulation, and loss of acinar and ductal parenchyma (4–6). These pathological abnormalities, for whose characterization SG biopsy is the gold standard technique, lead to the typical glandular inhomogeneity detected by salivary gland ultrasonography (SGUS), with hypo/anechoic areas and hyperechoic bands (7–10). So far, parenchymal inhomogeneity proved to be the main sonographic feature to build SGUS scores in pSS (7, 8, 11). In 1992, De Vita et al. firstly developed a comprehensive sonographic score of major SGs in pSS defining, by means of a discriminant analysis, inhomogeneity as the main SGUS abnormality associated with pSS; the developed semi-quantitative score ranged from 0 to 3 in each gland, from normal-appearing morphology to severe inhomogeneity (7). Several scoring systems have been proposed subsequently, and most of them used glandular inhomogeneity as the key SGUS abnormality. In addition, several clinicians now routinely use SGUS to assess patients with suspected or established pSS (9, 10, 12). However, even if many authors strongly believe that SGUS is relevant for the management of pSS, definite recommendations are still lacking, and this technique is not yet part of the classification criteria for pSS. This is mainly due to: (i) the absence of consensus on elementary SGUS lesions and scoring when pSS classification criteria were set up (12); (ii) the evidence of significant intra- and inter-rater disagreement (12–14); (iii) the use of old pSS cohorts for validation of pSS classification criteria, when SGUS was not yet fully developed (15). In order to overcome these issues, the Outcome Measure in Rheumatology (OMERACT) working group on the use of ultrasonography in pSS recently generated, after a three-round Delphi process, the definitions for the SGUS elementary lesions in pSS, and the scanning procedure (16). Lastly, the same OMERACT group developed a four-grade SGUS score, which showed excellent intra-rater reliability and a good inter-rater reliability between experts (16). By the possible addition of SGUS as a new criterion, the 2016 ACR-EULAR criteria for the classification of pSS may be ameliorated in sensitivity without modifying the specificity (17).

On the other hand, very few data exist to support the reliability of SGUS in pSS, and all the published studies involved only experts and well-trained sonographers. Therefore, it definitely remains to be investigated how reliability varies along with the observer training level and experience, and this still represents the major obstacle for a wider acceptance of SGUS in pSS evaluation (13).

Furthermore, the images from previous studies were not publicly available, making rather difficult for subsequent studies to reproduce and/or to objectively compare their findings. Accordingly, the leading pSS experts (35 partners from 13 countries) have recently started the HarmonicSS (http://harmonicss.eu) initiative to envelop independently reported cohorts and metacentric data, including SGUS in a dedicated Workpackage (WP), namely WP7. This study was created within the HarmonicSS initiative, and it is preliminary to further studies on the application of artificial intelligence in SGUS. At the current stage of the initiative, this study aims to: (i) evaluate the reliability among sonographers with different levels of expertise in SGUS; (ii) compare the reliability performance between two different semi-quantitative SGUS scores, widely used and easy to perform, being the “extremes” in the year of publication (i.e., the scores by De Vita et al., 1992, and by OMERACT, 2019); (iii) provide a data set that will be publicly available, to serve as a standardized benchmark for further studies.

## Materials and Methods

The Guidelines for Reporting Reliability and Agreement Studies (GRAAS) were followed for the preparation of the manuscript (18).

### Salivary Gland Ultrasound Scores

A simple, semi-quantitative 0–3 SGUS scoring system was recently selected by a systematic review and meta-analysis as more appropriate for diagnostic purposes in terms of specificity and heterogeneity in pSS, with respect to the other scoring systems available (e.g., 0–16 and 0–48) (19). One of the aims of the work-package 7 (WP7) of the HarmonicSS project is to develop and improve the role of SGUS for pSS management. Participants and coordinators of WP7 agreed to use two four-grade semi-quantitative scoring systems, namely De Vita et al. score (7) and OMERACT score (16), for the assessment of major SGs morphology in pSS patients enrolled in the HarmonicSS project.

**Figure 1:**
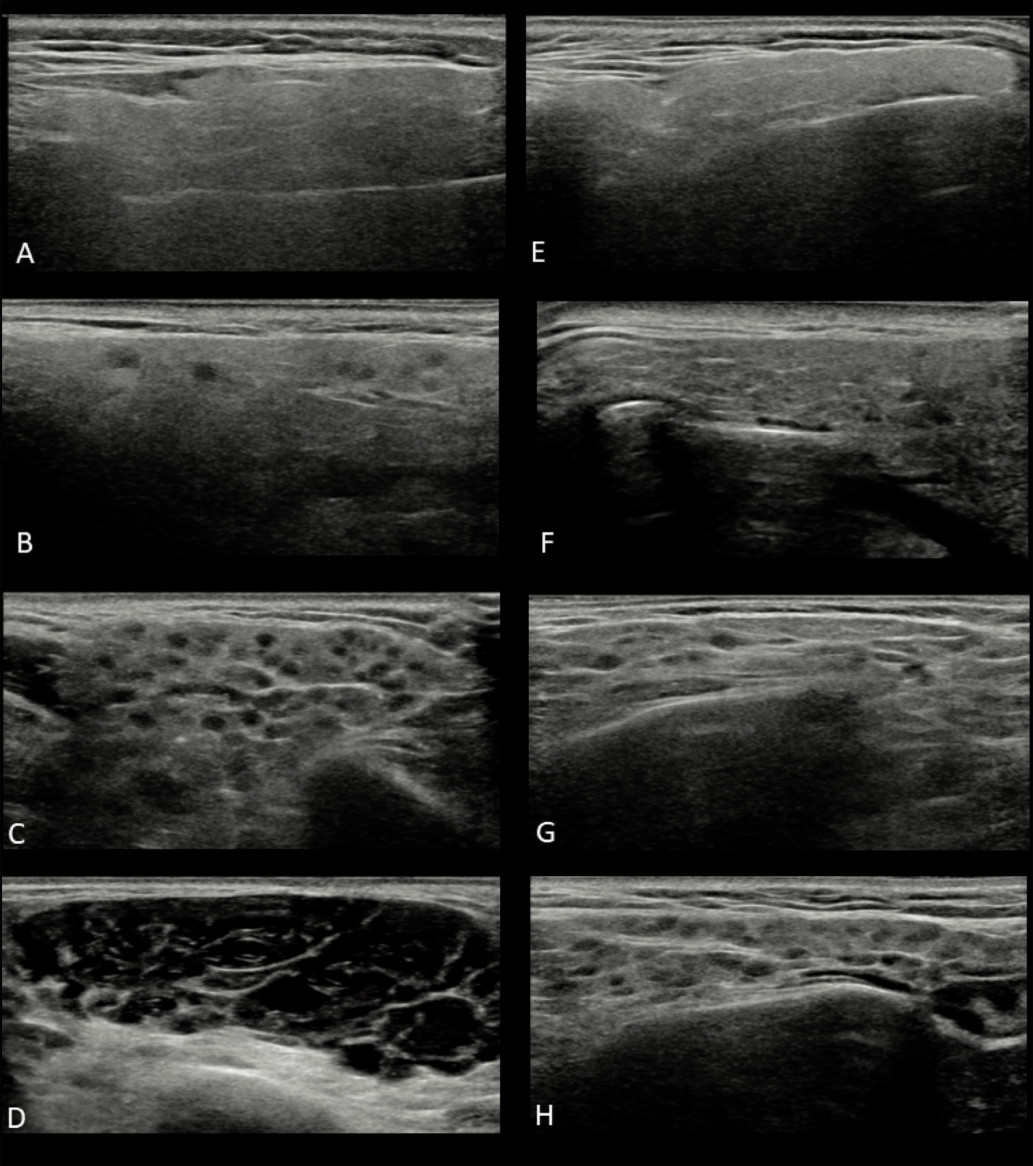
Ultrasound images of parotid glands in the two four-grade semi-quantitative scoring system: **(A)** De Vita et al. score grade 0; **(B)** De Vita et al. score grade 1; **(C)** De Vita et al. score grade 2; **(D)** De Vita et al. score grade 3; **(E)** OMERACT score grade 0; **(F)** OMERACT score grade 1; **(G)** OMERACT score grade 2; **(H)** OMERACT score grade 3.

The score by De Vita et al. is the long-standing available in the literature in pSS, is easy-to-perform, and includes both hypo/anechoic areas and hyperechoic bands as the main sonographic features to define parenchymal SG inhomogeneity, while in the OMERACT score parenchymal inhomogeneity is supported only by the presence of hypo/anechoic areas. The De Vita et al. score comprises: grade 0, normal-appearing parenchyma; grade 1, mild inhomogeneity with isolated and small hypo/anechoic areas, without hyperechoic bands; grade 2, evident inhomogeneity with multiple scattered hypo/anechoic areas and/or few hyperechoic bands; grade 3, severe/gross inhomogeneity due to large and confluent hypo/anechoic areas and/or diffuse hyperechoic bands (Figure 1). In this exercise, as well as in the recent studies where the De Vita et al. score was applied (20–22), the grade 1 was better specified, since the term of “mild inhomogeneity” was initially included, as a diffuse or localized micro-areolar structure. The OMERACT score is the most recent one, proposed in 2019 according to guidelines for selecting outcome measure instruments (OMI) (23) and it is defined as follows: grade 0, normal-appearing SG parenchyma; grade 1, minimal change: mild inhomogeneity without hypo/anechoic areas; grade 2, moderate change: moderate inhomogeneity with focal hypo/anechoic areas; grade 3, severe change: diffuse inhomogeneity with hypo/anechoic areas occupying the entire gland surface (Figure 1).

### Participants

Twenty-seven sonographers involved in the WP7 of the HarmonicSS project (https://www.harmonicss.eu/the-project/project-structure/) from nine countries in Europe (Austria, France, Germany, Italy, Norway, Serbia, Slovenia, The Netherlands, and United Kingdom) were invited to participate. The years of experience in SGUS, the number of pSS patients evaluated per year, and scores usually used in clinical practice were collected for each participant. Sonographers with at least 6 years of experience in SGUS were identified as experts, while the user definition was applied in those who already applied the De Vita et al. and/or OMERACT scores in clinical practice or for research purposes. Four different levels of sonographers were then identified as follows: user and experts (U-E group), non-users and experts (NU-E group), users and non-experts (U-NE), non-users, and non-experts (NU-NE group). A dedicated SGUS atlas was sent to all participants, with general SGUS issues, and with definitions and examples for each grade of glandular inhomogeneity for both scores.

### Reliability Exercise and HarmonicSS Data Set

A pool of 225 sonographic static images (83 normal-appearing images, 42 images with mild inhomogeneity, 47 images with moderate inhomogeneity, and 53 images with severe inhomogeneity) of major SGs [114 parotid glands (PGs) and 111 sub-mandibular glands (SMGs)], from 150 patients with suspected pSS or definite pSS, was independently scored in two rounds. The sonographic images were previously collected and de-identified from the database of four rheumatologists involved in the exercise (AZ, AH, VM, ODL) and were different from those presented in the atlas. Four different ultrasound machines were used to store images, i.e., Samsung RS85, Philips Epiq, GE Logiq E9, and ESAOTE MyLab70. For both scoring rounds, each observer was provided with an anonymized and uniquely randomized data set in order to ensure that scorings performed in this study could be not influenced with others' scorings. The reliability exercise was carried out remotely by using the HarmonicSS web-based platform (https://private.harmonicss.eu). For each round, the participants had to apply the De Vita et al. score and the OMERACT score for each of the 225 images. The described data set, together with accompanying script files and instructions for their usage, are publicly available on the GitHub repository (https://github.com/ArsoVukicevic/Assessment-of-pSS-fromSGUSimages/tree/master/3%20HarmonicSS%20benchmark%20dataset) that will be further managed by the HarmonicSS group and authors of this study.

### Statistical Analysis

Inter-rater reliability was assessed by using kappa statistics and computing the linear and squared weighted and unweighted kappa coefficient (Fleiss-Cohen weights) for each pair of raters for both scores considered. In the analyses the weighted kappa (i.e., linear and squared kappa), in addition to unweighted kappa, was performed since the use of weighting schemes allows to take into account the closeness of agreement between categories. The weights are presented in Supplementary Table 1. The mean, median, 1st and 3rd quartile, minimum (min), and maximum (max) kappa values were calculated. Then Light's kappa was considered as the mean kappa value. To assess intra-rater reliability, we computed the linear and squared weighted and unweighted kappa coefficients between two readings by each rater for both scores. We then computed Light's kappa (mean of intra-rater kappa values), median, 1st and 3rd quartile, minimum (min), and maximum (max) kappa values. The bootstrap percentile method was used to compute the 95% CI of every Light's, Fleiss and Cohen kappa. Furthermore, we converted every 0–3 score database in 0–1 score database considering scores 0 and 1 as normal-appearing scores and converted to 0; whereas scores 2 and 3 were held as pathological scores and were converted to score 1. Every analysis was repeated in the 0–1 score database. We stratified squared weighted kappa coefficients by levels of SGUS experience and the knowledge of the tested scoring systems. We obtained kappa for four levels combining experience and use information: U-E, U-NE, NU-E, and NU-NE. The results were then compared to evaluate the overlap between 95% confidence intervals. Kappa coefficients were interpreted according to Landis and Koch (24). Kappa values of 0–0.20 were considered slight, 0.21–0.40 fair, 0.41–0.60 moderate, 0.61–0.80 substantial, and 0.81–1.00 almost perfect. All statistical analyses were carried out using R version 3.6.2 (R Foundation for Statistical Computing, Vienna, Austria). For clarity, only weighted kappa are displayed in the text and tables; however, full statistical results are available in the Supplementary Material results and in the repository.

## Results

### Participants

Twenty out of 27 invited sonographers from eight countries in Europe (Austria, France, Germany, Italy, Norway, Serbia, Slovenia, and The Netherlands) participated in the two rounds of the reliability exercise. Seventeen out of 20 were rheumatologists, two were radiologists, and one was a specialist in oral medicine. Their experience with SGUS was 8.75 (± 5.97) years, with a mean number of pSS patients evaluated by SGUS per year of 127.6 (± 108.43). Thirteen out of 20 (65%) sonographers were experts, and 14/20 (70%) were users. The mean SGUS use was 11.8 (± 5.0) years for experts and 3.0 (± 1.5) years for non-experts (*p* < 0.001). Nine out of twenty (45%) sonographers were U-E, 5/20 (25%) were U-NE, 4/20 (20%) were NU-E, and 2/20 (10%) were NU-NE.

### Participants Scores

The mean De Vita et al. score and OMERACT score of the first round is reported in Supplementary Figure 1. The mean De Vita et al. score was 1.14 (± 1.10) for U-E, 1.33 (± 1.12) for U-NE, 1.31 (± 1.10) for NU-E, and 1.45 (± 1.08) for NU-NE. The mean OMERACT score was 1.28 (± 1.16) for U-E, 1.41 (± 1.07) for U-NE, 1.41 (± 1.13) for NU-E, and 1.80 (± 1.08) for NU-NE. The mean OMERACT score in NU-NE was significantly higher compared with U-E group (*p* < 0.05). No other significant differences among groups were present.

### Reliability Exercise

#### Intra-Rater Reliability

The intra-rater reliability for the De Vita et al. and the OMERACT score was almost perfect with a Light's Kappa of 0.86 (0.79–0.95; 95% CI 0.84–0.89) and 0.87 (range 0.81–0.95; 95% CI 0.85–0.90), respectively (Table 1). High levels of reliability were also reached when comparing normal-appearing scores vs. pathological scores, achieving a Light's Kappa of 0.80 for the De Vita et al. (0.67–0.95; 95% CI 0.76–0.84) and 0.81 for the OMERACT score (0.59–0.95; 95% CI 0.76–0.85) (Table 1). Unweighted kappa and weighted linear kappa are presented in Supplementary Table 2. No significant differences were found between the intra-rater reliability of PGs and SMGs (Supplementary Table 3).

**Table 1:** Intra-rater reliability.

**Kappa intra-rater**	**Light's kappa**	**Kappa min**	**Kappa max**	**95% CI**
De Vita et al. score 0–3 weighted (squared)	0.86	0.79	0.95	0.84–0.89
De Vita et al. score normal vs. pathological (0 vs. 1)	0.80	0.67	0.95	0.76–0.84
OMERACT score 0–3 weighted (squared)	0.87	0.81	0.95	0.85–0.90
OMERACT score normal vs. pathological (0 vs. 1)	0.81	0.59	0.95	0.76–0.85

#### Inter-Rater Reliability

The inter-rater reliability for the De Vita et al. and the OMERACT score was substantial with Light's Kappa of 0.76 (0.50–0.92; 95% CI 0.72–0.79) and 0.77 (0.57–0.93; 95% CI 0.74–0.80), respectively (Table 2). Comparing normal-appearing scores (0–1) vs. pathological scores (2–3), the Light's kappa value was substantial both for the De Vita et al. score (kappa value: 0.68; range 0.37–0.86; 95% CI 0.63–0.72) and the OMERACT score (kappa value: 0.71; range 0.42–0.92; 95% CI 0.65–0.75) (Table 2). Similar results were obtained in the second round of the exercise (Table 2). Unweighted kappa and weighted linear kappa of the inter-rater reliability are presented in Supplementary Tables 4, 5. Higher inter-rater reliability was achieved in PGs compared to SMGs (Table 3).

**Table 2:** Inter-rater reliability.

**Kappa inter-rater**	**Light's kappa**	**Kappa min**	**Kappa max**	**95% IC**
**I Round**				
De Vita et al. score 0–3 weighted (squared)	0.76	0.50	0.92	0.72–0.79
De Vita et al. score normal vs. pathological (0 vs. 1)	0.68	0.37	0.86	0.63–0.72
OMERACT score 0–3 weighted (squared)	0.77	0.57	0.93	0.74–0.80
OMERACT score normal vs. pathological (0 vs. 1)	0.71	0.42	0.92	0.65–0.75
**II Round**				
De Vita et al. score 0–3 weighted (squared)	0.73	0.45	0.91	0.70–0.76
De Vita et al. score normal vs. pathological (0 vs. 1)	0.67	0.29	0.89	0.62–0.71
OMERACT score 0–3 weighted (squared)	0.74	0.58	0.93	0.71–0.77
OMERACT score normal vs. pathological (0 vs. 1)	0.70	0.49	0.91	0.65–0.74

**Table 3:** Inter-rater reliability in the first and second round: comparison between parotid and submandibular glands.

	**Light's kappa**	**Kappa min**	**Kappa max**	**95% IC**
**I Round**				
PGs–De Vita et al. score 0–3 Kappa weighted (squared)	0.81^*^	0.60	0.94	0.77–0.85^*^
SMGs–De Vita et al. score 0–3 Kappa weighted (squared)	0.68	0.35	0.91	0.62–0.73
PGs–OMERACT score 0–3 Kappa weighted (squared)	0.81^*^	0.62	0.94	0.78–0.85^*^
SMGs–OMERACT score 0–3 Kappa weighted (squared)	0.70	0.50	0.92	0.65–0.75
**II Round**				
PGs–De Vita et al. score 0–3 Kappa weighted (squared)	0.77	0.54	0.91	0.73–0.80
SMGs–De Vita et al. *s*core 0–3 Kappa weighted (squared)	0.68	0.30	0.92	0.62–0.73
PGs–OMERACT score 0–3 Kappa weighted (squared)	0.76^*^	0.62	0.93	0.74–0.82^*^
SMGs–OMERACT score 0–3 Kappa weighted (squared)	0.69	0.45	0.93	0.63–0.73

*PGs, Parotid Glands; SMGs, Submandibular glands*.

* *Significant difference between the reliability of PGs and SMGs*.

#### Intra-Rater Reliability Among Sonographers With Different Levels of Experience and Use of SGUS Scores

The intra-rater reliability for the De Vita et al. score and the OMERACT score was almost perfect in all groups. For the De Vita et al. score, the Light's kappa ranged from 0.84 for U-NE (0.79–0.88; 95% CI 0.82–0.86) to 0.88 for NU-E (0.81–0.95; 95% CI 0.85–0.92). For the OMERACT score the Light's kappa ranged from 0.85 for U-NE (0.82–0.86; 95% CI 0.84–0.85) to 0.91 for NU-E (0.84–0.95; 95% CI 0.87–0.92) (Table 4).

**Table 4:** Intra-rater reliability among different group of sonographers.

**Intra-rater reliability among groups**		**Light's kappa**	**Kappa min**	**Kappa max**	**95% CI**
De Vita et al. score 0–3 weighted (squared)	U-E group	0.87	0.82	0.91	0.85–0.89
	U-NE group	0.84	0.79	0.88	0.82–0.86
	NU-E group	0.88	0.81	0.95	0.85–0.92
	NU-NE group	0.87	0.87	0.87	0.87–0.87
De Vita et al. score normal vs. pathological(0 vs. 1)	U-E group	0.82	0.74	0.87	0.79–0.85
	U-NE group	0.74	0.67	0.81	0.71–0.77
	NU-E group	0.85	0.78	0.95	0.81–0.89
	NU-NE group	0.80	0.78	0.82	0.79–0.81
OMERACT score 0–3 weighted (squared)	U-E group	0.87	0.83	0.93	0.86–0.90
	U-NE group	0.85	0.82	0.86	0.84–0.85
	NU-E group	0.91	0.84	0.95	0.87–0.92
	NU-NE group	0.85	0.81	0.89	0.82–0.87
OMERACT score normal vs. pathological(0 vs. 1)	U-E group	0.83	0.77	0.92	0.81–0.86
	U-NE group	0.78	0.75	0.82	0.76–0.80
	NU-E group	0.86	0.81	0.95	0.82–0.89
	NU-NE group	0.68	0.59	0.76	0.63–0.73

*U-E group, user and experts; U-NE group, users and non-experts; NU-E group, non-users and experts; NU-NE group, non-users and non-experts*.

#### Inter-Rater Reliability Among Sonographers With Different Levels of Experience and Use of SGUS Scores

The Light's kappa of the four groups showed a substantial level of inter-reliability for the De Vita et al. and the OMERACT scores in both rounds (Table 5). For the De Vita et al. score, the Light's kappa of the first round ranged from 0.71 for U-NE (0.66–0.75) to 0.79 for NU-NE (0.75–0.84). For OMERACT score the Light's kappa of the first round ranged from 0.78 for U-E (0.75–0.81), U-NE (0.74–0.81), and NU-E (0.74–0.82) to 0.79 for NU-NE (0.73–0.84) (Table 5).

**Table 5:** First and second round inter-rater reliability among different groups of sonographers.

**Inter-rater reliability among groups**		**Light's kappa**	**95% CI**
I round De Vita et al. score 0–3 weighted (squared)	U-E group	0.78	0.74–0.81
	U-NE group	0.71	0.66–0.75
	NU-E group	0.77	0.72–0.80
	NU-NE group	0.79	0.75–0.84
II round De Vita et al. score 0–3 weighted (squared)	U-E group	0.74	0.70–0.87
	U-NE group	0.70	0.66–0.75
	NU-E group	0.75	0.70–0.79
	NU-NE group	0.77	0.72–0.81
I round OMERACT score 0–3 weighted (squared)	U-E group	0.78	0.75–0.81
	U-NE group	0.78	0.74–0.81
	NU-E group	0.78	0.74–0.82
	NU-NE group	0.79	0.73–0.84
II round OMERACT score 0–3 weighted (squared)	U-E group	0.73	0.69–0.77
	U-NE group	0.72	0.67–0.75
	NU-E group	0.78	0.74–0.81
	NU-NE group	0.85	0.81–0.89

*U-E group, user and experts; U-NE group, users and non-experts; NU-E group, non-users and experts; NU-NE group, non-users and non-experts*.

## Discussion

In this web-based reliability exercise, two different semi-quantitative SGUS scores for pSS proved to be reliable for the sonographic evaluation of the major SGs. In addition, regardless of the level of the sonographer's experience, an almost perfect intra-rater reliability, and substantial inter-rater reliability, were reached. Finally, images and data of the present study will be publicly available to facilitate further investigations.

Few previous studies evaluated the reproducibility of SGUS in pSS, usually with few experts and with variable results (8, 9, 11). Recently the authors of the OMERACT score of SGUS in pSS highlighted a good reliability for their score among 25 experts (16). This study involved an equally relevant number of raters, but also with different levels of expertise. In order to use SGUS as an OMI and as an item for pSS classification, its reliability must be tested, and it is recommended that the weighted kappa should be >0.7 (25). Over the past years, SGUS has received a growing interest as it is a non-invasive and easily performed technique for the management of pSS (26). Furthermore, in clinical practice, SGUS semi-quantitative scores are easy to apply and have a good discriminatory power between pathological and normal-appearing major SGs (7, 8, 10). This exercise tested the application of two different, easy-to-apply, 0–3 semi-quantitative scores for SGUS, namely the ones developed by De Vita et al. in 1992 and by OMERACT in 2019. The former includes features of both inflammation (i.e., hypo/anechoic areas) and damage (i.e., hyperechoic bands), whereas the latter, including mainly features of glandular inflammation, was built following the recent OMI recommendations. In this study, both the scores showed an almost perfect intra-rater reliability and substantial inter-rater reliability among 20 sonographers with different levels of experience and knowledge of SGUS scores. The number of involved sonographers and the stratification of the sonographers, based on their experience, are the main strengths of this study. In this reliability exercise, being a non-expert and/or a non-user did not significantly impact the level of agreement among raters. Importantly, however, a support for SGUS evaluation and scoring was given to raters, by means of a dedicated atlas of images. We did not investigate whether the less expert sonographers were those mainly using the atlas or not, since it was poorly feasible. Further studies, in any case, should better define the optimal way to support SGUS rating in pSS. The automatic scoring of SGUS by image segmentation and artificial intelligence is also being evaluated in HarmonicSS.

As already highlighted by the OMERACT study, the reliability among PGs and SMGs was different also in this study, and worse in the SMGs than in the PGs for both the tested scores (16). This could be in part expected since a mild inhomogeneity (e.g., grade 1 for both the scores) of the SMGs can be difficult to be differentiated from the normal gland. The main limitation of the study was the absence of a patient-based exercise; ideally, reliability testing should also be performed in the clinical setting with the patient, and not with the sole images. In this scenario, practical sonographic skills could make the difference among groups with different levels of experience, but a high number of sonographers are needed, making this type of exercise challenging to plan. Furthermore, the presence of only two raters in the NU-NE group could be another study limitation. In this multi-center reliability exercise, groups with an equal number of participants could not be defined a priori, since the choice of sonographers was made by each center involved in the European project.

In conclusion, this study focused on SGUS reliability, i.e., the main limit for a wider use of SGUS in pSS. Both the tested SGUS scores proved to be reliable for the evaluation of pSS patients and this reinforces and supports the reliability of SGUS as highlighted by the OMERACT study (16). Furthermore, in this study the agreement was independent of the years of experience of sonographers and of their previous use of the tested scores. Overall, in our opinion, major evidence to further encourage the use of SGUS in pSS in the next future is provided.

## Patient and Public Involvment in Research

EULAR PARE and SSF–Sjögren's Syndrome Foundation have an advisory role in HarmonicSS and will continuously monitor and evaluate the project (outcomes) in terms of impact to patients. Also, EULAR PARE and SSF have a major role in the dissemination of the results to patient associations and the public. https://www.harmonicss.eu/patients-advisory-group/.

## Data Availability Statement

The raw data supporting the conclusions of this article will be made available by the authors, without undue reservation.

## Ethics Statement

The studies involving human participants were reviewed and approved by CEUR-2017-Os-027-ASUIUD. The patients/participants provided their written informed consent to participate in this study.

## Author Contributions

AZ, SZ, AT, AV, AH, VM, GC, MC, KD, OD, DE, FF, AG, GG, IG, DH, MJ, SJ-J, PM, SP, CP, MS, NF, AGT, FV, and SD: conception and design. AZ, AT, and FV: statistical analysis. AZ, SZ, and SD: wrote the manuscript and interpretation of the results. All authors: contributed to the article and approved the submitted version.

## Conflict of Interest

The authors declare that the research was conducted in the absence of any commercial or financial relationships that could be construed as a potential conflict of interest. The handling editor declared a shared affiliation, though no other collaboration, with the authors GC and CP.

## Abbreviations

ACR-EULAR: American College of Rheumatology/European League Against Rheumatism
GRAAS: Guidelines for Reporting Reliability and Agreement Studies
NU-E: non-users and experts
NU-NE: non-users and non-experts
OMERACT: Outcome Measures in Rheumatology Clinical Trials
OMI: outcome measure instruments
PG: parotid gland
pSS: primary Sjögren's syndrome
SGs: salivary glands
SGUS: salivary gland ultrasound
SMG: submandibular gland
U-E: user and experts
U-NE: users and non-experts.
