# Supplementary material for: Salivary Gland Ultrasonography in Sjögren's Syndrome: A European Multicenter Reliability Exercise for the HarmonicSS Project
Source: Front Med (Lausanne). 2020 Nov 23;7:581248. doi: 10.3389/fmed.2020.581248 (PMC7719819; doi:10.3389/fmed.2020.581248)

| **Difference** | **Linear weights** | **Squared weights** |
| --- | --- | --- |
| **0** | **1** | **1** |
| **1** | **0.66** | **0.89** |
| **2** | **0.33** | **0.55** |
| **3** | **0** | **0** |

**Table S1. The weights used for calculation of weighted kappa.**

**Table S2.** Intra-rater reliability.

| Kappa intra-rater  De Vita *et al.* Score | De Vita *et al.* score  normal vs pathological (0 vs 1) | De Vita *et al*. score  0-3 unweighted | De Vita *et al*. score 0-3 weighted (linear) | De Vita *et al*. score  0-3 weighted (squared) | Kappa intra-rater  OMERACT Score | OMERACT score  normal vs pathological (0 vs 1) | OMERACT score  0-3 unweighted | OMERACT score 0-3 weighted (linear) | OMERACT score  0-3 weighted (squared) |
| --- | --- | --- | --- | --- | --- | --- | --- | --- | --- |
| Kappa | 0.80 | 0.68 | 0.78 | 0.86 | **Kappa** | 0.81 | 0.67 | 0.78 | 0.87 |
| Kappa min | 0.67 | 0.53 | 0.70 | 0.79 | **Kappa min** | 0.59 | 0.46 | 0.66 | 0.81 |
| Kappa max | 0.95 | 0.95 | 0.95 | 0.95 | **Kappa max** | 0.95 | 0.95 | 0.95 | 0.95 |
| 95% CI | 0.76 – 0.84 | 0.62 - 0.74 | 0.75 - 0.82 | 0.84 - 0.89 | **95% CI** | 0.76 - 0.85 | 0.61 - 0.73 | 0.75 – 0.82 | 0.85 – 0.90 |

**Table S3.** Intra-rater reliability for the parotid glands (PGs) and submandibular glands (SMGs) evaluation.

|  | **Light’s kappa** | **Kappa min** | **Kappa max** | **95% CI** |
| --- | --- | --- | --- | --- |
| **PGs - De Vita *et al*. score 0-3**  **Kappa weighted (squared)** | 0.88 | 0.78 | 0.99 | 0.83 - 0.89 |
| **SMGs –De Vita *et al.* score 0-3**  **Kappa weighted (squared)** | 0.84 | 0.72 | 0.93 | 0.80 - 0.87 |
| **PGs - OMERACT score 0-3**  **0-3 weighted (squared)** | 0.89 | 0.83 | 0.97 | 0.87 - 0.91 |
| **SMGs – OMERACT score 0-3**  **Kappa weighted (squared)** | 0.84 | 0.72 | 0.94 | 0.80 - 0.88 |

**Table S4.** Inter-rater reliability of the De Vita *et al.* score.

| **Kappa inter-rater**  **ROUND 1** | **De Vita *et al. s*core**  **normal vs pathological (0 vs 1)** | **De Vita *et al*. score 0-3 unweighted** | **De Vita *et al*. score 0-3 weighted (linear)** | **De Vita *et al*. score 0-3 weighted (squared)** | **Kappa inter-rater**  **ROUND 2** | **De Vita *et al. s*core**  **normal vs pathological (0 vs 1)** | **De Vita *et al*. score 0-3 unweighted** | **De Vita *et al*. score 0-3 weighted (linear)** | **De Vita *et al*. score 0-3 weighted (squared)** |
| --- | --- | --- | --- | --- | --- | --- | --- | --- | --- |
| **Kappa** | 0.68 | 0.44 | 0.61 | 0.76 | **Kappa** | 0.67 | 0.40 | 0.59 | 0.73 |
| **Kappa min** | 0.37 | 0.11 | 0.32 | 0.50 | **Kappa min** | 0.29 | 0.08 | 0.27 | 0.45 |
| **Kappa max** | 0.86 | 0.74 | 0.85 | 0.92 | **Kappa max** | 0.89 | 0.74 | 0.84 | 0.91 |
| **95% CI** | 0.63 – 0.72 | 0.40 – 0.47 | 0.57 – 0.65 | 0.72 – 0.79 | **95% CI** | 0.65 – 0.74 | 0.37 – 0.44 | 0.55 – 0.62 | 0.70 – 0.76 |

**Table S5.** Inter-rater reliability of the OMERACT score.

| **Kappa inter-rater**  **ROUND 1** | **OMERACT score**  **normal vs pathological (0 vs 1)** | **OMERACT**  **score 0-3 unweighted** | **OMERACT score 0-3 weighted (linear)** | **OMERACT score 0-3 weighted (squared)** | **Kappa inter-rater**  **ROUND 2** | **OMERACT score**  **normal vs pathological (0 vs 1)** | **OMERACT score 0-3 unweighted** | **OMERACT score 0-3 weighted (linear)** | **OMERACT score 0-3 weighted (squared)** |
| --- | --- | --- | --- | --- | --- | --- | --- | --- | --- |
| **Kappa** | 0.71 | 0.43 | 0.62 | 0.77 | **Kappa** | 0.70 | 0.41 | 0.60 | 0.74 |
| **Kappa min** | 0.42 | 0.10 | 0.38 | 0.57 | **Kappa min** | 0.49 | 0.11 | 0.37 | 0.58 |
| **Kappa max** | 0.92 | 0.78 | 0.87 | 0.93 | **Kappa max** | 0.91 | 0.78 | 0.87 | 0.93 |
| **95% CI** | 0.65 – 0.75 | 0.40 – 0.46 | 0.58 – 0.65 | 0.74 – 0.80 | **95% CI** | 0.65 – 0.74 | 0.38 – 0.44 | 0.56 – 0.62 | 0.71 – 0.77 |

**Figure S1.** The mean De Vita *et al.* score and OMERACT score among the different groups of sonographers.


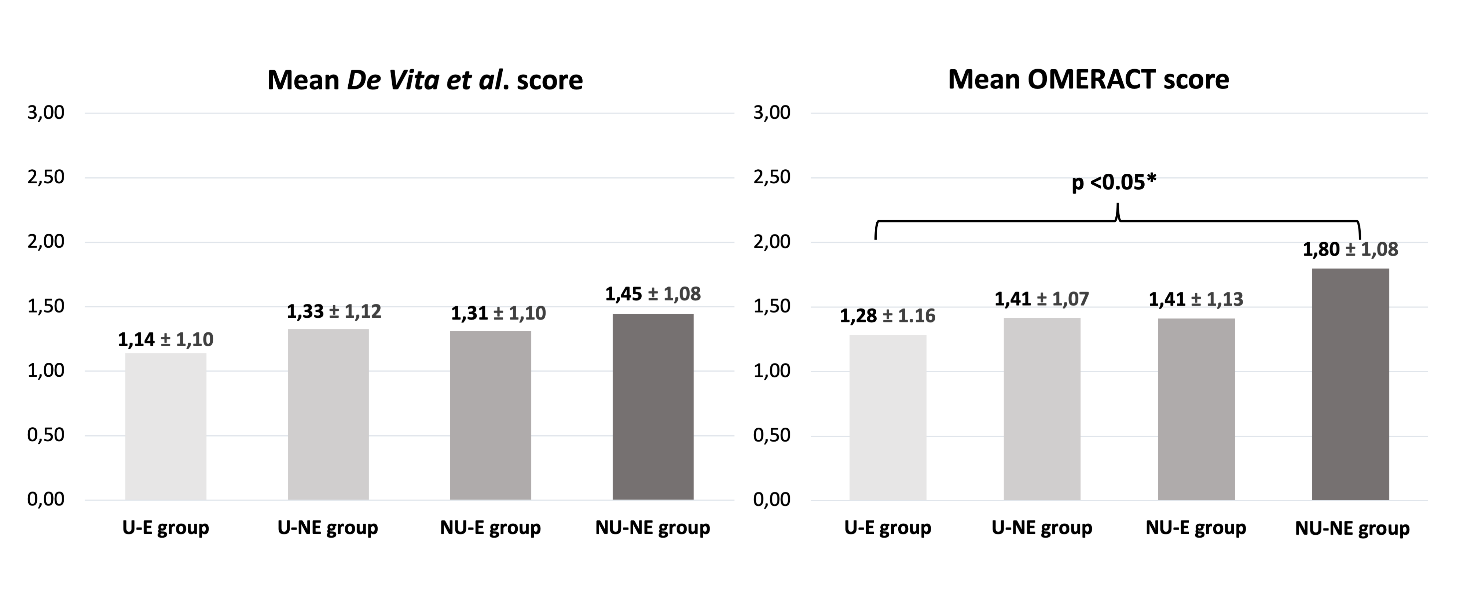

Supplement: Supplementary file 1 [file Data_Sheet_1.docx]
